# Pay and Allowances of the Medical Reserve Corps Members and Other Service Conditions

**Published:** 1917-08

**Authors:** 


### PAY AND ALLOWANCES OF THE MEDICAL RESERVE CORPS MEMBERS AND OTHER SERVICE CONDITIONS.

### Acceptance of Commission Is for Five Years Unless Sooner Relieved—Can Practice Specialty When Feasible.

The subcommittee on ophthalmology and oto-laryingology of the general medical board, Council of National Defense, in reply to inquiries from physicians, has sent the following:

Dear Doctor: In reply to your letter of inquiry, the subcommittee on ophthalmology and oto-laryngology refers you to the following:

1. Officers of the Medical Reserve Corps who have specialized in medicine or surgery will be given an opportunity to perform the duties of their specialty when feasible, men of larger experience naturally being given preference.

2. In the Medical Reserve Corps there are three grades, viz, lieutenant, captain, and major. It is the policy of the Surgeon General's Office to recommend the great majority of applicants for commission in the grade of first lieutenant, with the expectation of making numerous promotions when the officers concerned have had an opportunity to demonstrate their professional qualifications and their adaptability to the military service after a reasonable period of active duty. Applications for increased grade are not favorably considered unless they come through military channels, in order that the Surgeon General may have the benefit of the recommendations made by the applicant's superior officers. Political influence is unnecessary.

### The Chief Considerations.

In making recommendations for original commission age, professional attainments, and previous military experience are the chief considerations in determining the grade in which the applicant should be commissioned.

3. The pay of the different grades is: First Lieutenant, $2,000; captain, $2,400; major, $3,000.

When assigned to duty in a city (not in camp, thus not serving the troops) the assigment carries with it commutation of quarters: First lieutenant, three rooms; captain, four rooms; major, five rooms; at $12 per room, heat and light additional.

4. Acceptance of a commission in the Medical Reserve Corps automatically places your services at the disposal of the Surgeon General wherever he deems them most valuable, either in the United States or abroad.

**Commissioned for Five Years.**

5. Acceptance of commission is for five years, unless sooner relieved from active duty on recommendation of the Surgeon General, when officers will be placed on the inactive list. Active duty in the present instance will naturally be for the length of the war plus four months, which will be required for the necessary physical examinations to be made of the men before they are discharged from the Army. The old requirement of three years' service including at least 90 days' active service before being elegible for promotion, has been eliminated.

6. In case of death from causes in line of duty, the Government pays to the widow or designated beneficiary six months' pay of the grade held by the deceased at the time of death. The deceased family is also entitled to a pension.

7. The limited numbers of quarters at the majority of Stations and campus makes it inadvisable for officers of the Reserve Corps to be accompanied by their families unless they can provide for them independently.

8. In no event will the families of officers be allowed to accompany them abroad.

9. Officers in the Medical Reserve Corps under the age of 45 years will be called for training in the medical officers' training camps. This is for the purpose of giving intensive training in administrative duties, a requirement for military service. Men over 45 years, if they so select, may attend a medical officers' training camp. If a surgeon has had military training, he may be called, without camp instruction, for active duty.

**Suggestions by Surgeon General.**

The following paragraphs are added from a letter from the Surgeon General to the chairman of the State committees of the medical section, Council of National Defense:

"1. It is believed that it would be of great advantage to this department if each State committee would make a census of its State, with a view of dividing the medical profession into two classes: (a) those who can not be spared for Army service because of their importance to the civil community, and (b) those who can be spared. Class (a) should be requested to refrain from offering their services. Class (b), on the contrary, should be encouraged promptly to apply for appointment. This office is frequently called upon to give advice along these lines in individual cases, but the department does not care to assume this responsibility, believing as it does that the question is one that can much better be decided by the State committee, acting in conjunction with the county committees.

"2 The department will not feel called upon to consult the list prepared under paragraph 1 when individual applications are received, since it will be assumed in all cases that the individual offering himself can be spared and will be at the disposal of the department for such duty as the exigencies of the service may demand.

"3 For the purpose of this census the State committee should act as a clearing house for the county committees.

**Practice in Specialties.**

"4 Frequently inquiries are made as to whether a medical officer will be assigned to duty in accordance with his medical specialty. In this connection attention is invited to the fact that a large proportion of the administrative work of the Medical Department of the newly organized Army will fall upon the officers of the medical Reserve Corps. The officers of the regular establishment are so few in number that they will be available for only the most important administrative positions. With this in mind, it will be readily understood that officers of the Reserve Corps must largely supplement their technical knowledge by a clear conception of military co-ordination and administration before they can be of the greatest service to the department.

"5. They should offer themselves without reservation, considering their medical training as the basis upon which to build their education as medical officers.

**All Card Indexed.**

"6. It is true, nevertheless, that all officers of the Reserve Corps are card indexed according to their special qualifications and that when the Army is fully organized and working smoothly every effort will be made to assign each officer where his special qualifications will be most useful to the government and where the work will be congenial to the officer himself.

"7 The department has at its command at present only about one-fourth the number of officers that will be required for an Army of 2,000,000 men. By the application of the selective draft the full quota can probably be raised without great difficulty. It will be more creditable to the profession, however, to attain this end by voluntary offer of service.

"8. A great deal of inconvenience has been caused those applying for appointment in the Medical Reserve Corps by reason of the delay in issuing commissions. The business of the War Department has expanded so rapidly that it has been impossible to secure the necessary additional assistance required to handle the work. The delay has occurred in this office as well as in the office of The Adjutant General. This condition is being remedied as fast as circumstances permit.

"9. Your committee is urged to take such steps as may secure the prompt acceptance of commission issued or their immediate return to this office."

Sincerely Yours,

Subcommittee on Ophthalmology and Oto-Laryngology,

General Board. Aug. 15, 1917.

